# Inhibition of hypoxic response decreases stemness and reduces tumorigenic signaling due to impaired assembly of HIF1 transcription complex in pancreatic cancer

**DOI:** 10.1038/s41598-017-08447-3

**Published:** 2017-08-11

**Authors:** Olivia McGinn, Vineet K. Gupta, Patricia Dauer, Nivedita Arora, Nikita Sharma, Alice Nomura, Vikas Dudeja, Ashok Saluja, Sulagna Banerjee

**Affiliations:** 1 Division of Basic and Translational Research, Department Of Surgery, University of Minnesota, Minneapolis, MN USA; 2 Department of Surgery, University of Miami, FL Miami, 33136 USA

## Abstract

Pancreatic tumors are renowned for their extremely hypoxic centers, resulting in upregulation of a number of hypoxia mediated signaling pathways including cell proliferation, metabolism and cell survival. Previous studies from our laboratory have shown that Minnelide, a water-soluble pro-drug of triptolide (anti-cancer compound), decreases viability of cancer cells *in vitro* as well as *in vivo*. However, its mechanism of action remain elusive. In the current study we evaluated the effect of Minnelide, on hypoxia mediated oncogenic signaling as well as stemness in pancreatic cancer. Minnelide has just completed Phase 1 trial against GI cancers and is currently awaiting Phase 2 trials. Our results showed that upon treatment with triptolide, HIF-1α protein accumulated in pancreatic cancer cells even though hypoxic response was decreased in them. Our studies showed even though HIF-1α is accumulated in the treated cells, there was no decrease in HIF-1 binding to hypoxia response elements. However, the HIF-1 transcriptional activity was significantly reduced owing to depletion of co-activator p300 upon treatment with triptolide. Further, treatment with triptolide resulted in a decreased activity of Sp1 and NF-kB the two major oncogenic signaling pathway in pancreatic cancer along with a decreased tumor initiating cell (TIC) population in pancreatic tumor.

## Introduction

Pancreatic Ductal Adenocarcinoma (PDAC) is among the most notorious cancers with a poor 5-years survival rate of 7%^1^. Lack of early detection, extreme metastatic potential and resistance to therapy are considered to be the main reasons for such dismal statistics. One of the main characteristics of pancreatic tumors which contribute to its chemoresistance phenotype is the extensive desmoplastic stroma^2, 3^. Recent research has shown that the extracellular matrix, comprising of mostly collagen and hyaluronic acid (HA) exert an interstitial pressure on the tumor vasculature, thereby compressing the blood vessels and preventing the drug delivery to the tumors^4, 5^. Studies have also revealed that once the stroma is depleted, the blood vessels become functional, which in turn results in better delivery of chemotherapeutic drugs to the tumor^5, 6^.

In addition to preventing efficacious delivery of chemotherapeutic compounds to the tumor, the robust stroma in pancreatic cancer also results in generation of hypoxic niches within the tumor, which trigger activation of hypoxia mediated pro-survival pathways^7, 8^. As a result, pancreatic tumors are notoriously hypoxic^9^. The hypoxic microenvironment results in activation of the transcription factor HIF-1 and HIF-2 (Hypoxia Inducible Factor 1 and 2). HIF is a heterodimer composed of one αand one β subunit that bind to DNA at the hypoxia responsive element (HRE) located in the promoter region of numerous genes to regulate their transcription under hypoxia. Under normal oxygen concentration, HIF-1α is degraded rapidly by a ubiquitin-proteasome mediated pathway. This is facilitated by hydroxylation of the two proline residues by the enzyme prolyl hydroxylase (PHD1,2 or 3) in the presence of co-factors α ketoglutarate (αKG) and iron (Fe^2+^). Hydroxylated HIF-1α is recognized by VHL (Von Hippel Lindau), which in turn assembles the E3 ligase complex. Poly-ubiquitination of HIF-1α and targets it to the proteasome for degradation. Thus, under normal oxygen concentration, the alpha subunit of HIF1 is degraded. Under hypoxic conditions, enzymatic activity of PHDs is inhibited owing to less production of TCA cycle intermediate αKG. This results in attenuated hydroxylation of HIF-1α, leading to its accumulation^10^.

In addition to activating a pro-survival signaling pathway within a tumor, hypoxia also drives stemness^11–13^ and chemoresistance within a tumor^14–16^. Previously published studies from our lab confirm this in pancreatic cancer cells as well^17^. Our studies have shown that hypoxia leads to accumulation of CD133^+^ stem-like tumor initiating cells in MIA PaCa-2 cells that lack this population under normoxic conditions^17^. Thus targeting hypoxia can lead to not only downregulation of pro-survival pathways, but may also affect tumor initiating populations, decreasing tumor recurrence and chemoresistance.

Previously published data from our laboratory have shown that Minnelide, a pro-drug of triptolide is effective against pancreatic cancer in preclinical evaluation^18^. In addition to having anti-tumor effects, Minnelide was also effective as an anti-stromal agent^6^. Our results showed that treatment with Minnelide relieved the interstitial pressure on the blood vessels resulting in more functional and “open” blood vessels^6^. However, in spite of a number of translational studies on this drug, its mechanism of action is still being unravelled. Several studies from our laboratory have explored multiple pathways affected by Minnelide in a number of cancers. However, a comprehensive mechanism of action of the drug is yet to be elucidated. Minnelide has shown promise in a recently concluded Phase 1 trial for advanced GI malignancies and is currently awaiting a Phase 2 trial in pancreatic cancer^19^. In this context, it is important to understand the mechanism by which Minnelide induces pancreatic tumor regression.

In the current study we thus evaluated the mechanism by which Minnelide decreased hypoxia response within tumors by dampening the HIF-1α mediated pro-survival signaling and decreased the population of tumor initiating cells in pancreatic cancer. Our results showed that treatment with Minnelide resulted in accumulation of HIF-1α within the pancreatic tumor (in both patient derived xenografts as well as in a spontaneous mouse model KRAS^G12D^TP53^R172H^PDX^Cre^ or KPC for pancreatic cancer) even though the hypoxia and hypoxia mediated signaling was decreased. Our results further indicated that *in vitro*, while Minnelide did not affect the DNA binding property of HIF-1α, it prevented the assembly of the transcription complex of HIF-1α by downregulating the expression of p300, thereby decreasing the transcriptional activity of HIF-1α. This led to the inhibition of pro-survival signaling in pancreatic cancer cells eventually leading to death.

## Methods

### Cell culture

MIA PaCa-2 cells (ATCC) were cultured in DMEM medium (Hyclone) supplemented with 10% FBS and 1% penicillin streptomycin. S2-VP10 cells were cultured in RPMI 1640 medium (Hyclone) with 10% FBS and 1% penicillin streptomycin. Triptolide was used at a concentration of 50 nM in all *in vitro* experiments based on the response of this compound from our previous studies^18, 20, 21^ (unless otherwise mentioned). The hypoxia mimetic cobalt chloride (CoCl_2_) (Sigma) was used at a concentration of 200 uM for HIF-1α reporter assays. The proteasome inhibitor MG-132 (Sigma) was used at a concentration of 10 uM for HIF-1α reporter assays. BAY87-2243, a hypoxia inhibitor was used in indicated concentration. Minnelide, the pro drug of triptolide, was used *in vivo* at indicated concentrations.

### Western blotting

Pancreatic tumor tissue or cultured pancreatic cancer cells were lysed using RIPA buffer (Boston Bioproducts) and protein concentration was estimated using the BCA protein estimation assay (Thermo Scientific). Protein levels were detected using anti-HIF-1α antibody (Novus Biologicals) and anti-beta-Actin antibody (Santa Cruz).

### Quantitative Real-time PCR

Quantitative real-time PCR for HIF-1α, MCT4, and PFKB was performed using primers from Qiagen (QuantiTect primer assay). Primers for VEGF were obtained from Life Technologies. RNA was isolated from cells according to the manufacturer’s instructions using Trizol (Invitrogen). The High Capacity cDNA Reverse Transcription Kit (Life Technologies) was used to convert 2 ug of RNA to cDNA according to manufacturers instruction. Sybr Green from Roche (Lightcycler 480 SYBR Green I Master) was used for real-time PCR and was performed on the Light Cycler II (Roche) according to manufacturer’s instruction.

### Dual Luciferase Reporter Assay for transcriptional activity of HIF-1α

HIF-1α activity assays were done using the Cignal reporter assay kit for HIF-1α (Qiagen). Transfections were performed using Attractene transfection reagent (Qiagen) according to the manufacturer’s instructions. The pancreatic cancer cells were treated approximately 24 hours after transfection and collected 24 hours after treatment. The activity was analyzed using the Dual-Luciferase reporter assay system (Promega) according to manufacturer’s instruction.

### HIF-1α DNA Binding Activity Assay

μHIF-1α DNA binding activity was assessed using the HIF-1α Transcription Factor Assay Kit (Cayman Chemical) on pancreatic cancer cells. Briefly, a dsDNA sequence containing the HIF-1α response element (5′-ACGTG-3′) is immobilized to the wells of a 96-well plate. Protein extracts from cells or tumors were equilibrated in the wells and active HIF-1α was free to bind to the HIF-1α response element. After washing, HIF-1α was detected using anti-HIF-1α antibody followed by a secondary antibody conjugated to HRP. The results were analyzed by measuring the absorbance at 450 nm. The samples were normalized by their relative protein concentration.

### Spontaneous Pancreatic Cancer Mouse Model

KRAS^G12D^; Trp53^R172H^; Pdx-1^Cre^ (KPC) mice were used for a spontaneous pancreatic cancer mouse model to study hypoxia in tumor progression and the effect of Minnelide on hypoxia and HIF-1α. For the tumor progression study, KPC mice ages 1 month, 3 month, and 6 month (4-5 mice per group) were sacrificed and the pancreata were collected. Pimonidazole (Hypoxyprobe)^22^ was injected (60 mg/kg body weight) intraperitoneally 4 hours prior to sacrifice.

For the Minnelide study, 10 3-month old KPC mice were injected with Minnelide (0.21 mg/kg body weight) intraperitoneally 5 days-on and 2 days-off for 38 days. 10 age matched KPC mice were injected with saline. Pimonidazole was administered 4 hours prior to sacrifice as described. All procedures were approved by the University of Minnesota Institutional Animal Care and Use Committee. All procedures were carried out in accordance with the above guidelines and regulations.

### Patient Derived Xenograft Mouse Model

Pancreatic tumors removed from de-identified patients were transplanted subcutaneously into SCID mice. The mice were treated with Minnelide (0.42 mg/kg body weight) for one month. The tumors were collected and HIF-1α protein levels were analyzed. All approvals were obtained from the University of Minnesota Institutional Review Board. Informed consent from all subjects was obtained according to guidelines. All procedures involving implanting patient tumor derived xenografts in mice were performed according to the approval guidelines of the University of Minnesota Institutional Animal Care and Use Committee.

### Immunohistochemistry

Paraffin embedded tissue sections (from patient tumor derived xenografts or KPC tumors) were received mounted on slides. The slides were deparaffinized in xylene and rehydrated. Antigen retrieval was performed by steaming slides with Target retrieval Solution, pH 9 (Dako). Serum Free Block (Dako) was used for blocking and Background Sniper (Biocare Medical) was used for antibody incubation. HIF-1α was detected using anti-HIF-1α antibody using a dilution of 1:100. Pimonidazole was detected using anti-pimonidazole antibody (Hypoxyprobe) at a dilution of 1:50. The slides were mounted using ProLong Gold Antifade Reagent with DAPI (Molecular Probes).

### Isolation of CD133+ tumor initiating cells

The CD133^+^ population was separated from pancreatic cancer cell S2-VP10 using MACS separation (Miltenyi Biotech) using manufacturers protocol. Cultured cells was bound to anti-mouse CD133-Microbeads for 10 min on ice and positively purified for CD133^+^ cells by MACS. The purity of separation was tested for each batch by performing a FACS analysis using Anti-CD133-PE antibody AC141 (Miltenyi Biotech). The separated populations were used for RNA, Protein and FACS analysis. Cells growing in culture were scraped gently into centrifuge tube and washed once in Wash Buffer (PBS, 0.5% BSA, 2 mM EDTA) before binding to Anti-mouse CD133 microbeads and proceeding as described above.

### Glucose Uptake assay

Cell based glucose uptake assay kit (Cayman Chemicals) was used for measuring glucose uptake in pancreatic cancer cells MIA-PACA2 and S2-VP10. For labeling, isolated cells were starved in glucose free media for 30 min prior to labeling with 150ug/ml 2-NBDG, a fluorescent analog of deoxyglucose. Following incubation for 1 h, the labeled cells were analyzed by flow-cytometry according to manufacturer’s instruction.

### Hydroxyprolination assay

Hydroxyproline assay was performed on pancreatic cancer cell and tumor lysate after treatment with triptolide for 24 h using a Hydroxyproline assay kit (Sigma) and results were expressed as Hydroxyproline formed per ug protein. Assay was performed according to manufacturer’s instruction.

### Estimation of alpha ketoglutarate and fumarate

Alpha ketoglutarate and fumarate were estimated from pancreatic cancer cell and tumor lysate using assay kits (Sigma) according to manufacturer’s instruction.

### Statistical Analysis

Values are expressed as the mean ± standard error. All experiments were performed at least three times. The significance of the difference between two samples was determined with an unpaired Student’s t-test. P-values of less than 0.05 were considered statistically significant.

## Results

### Pancreatic tumors are hypoxic in nature

**Figure 1:**
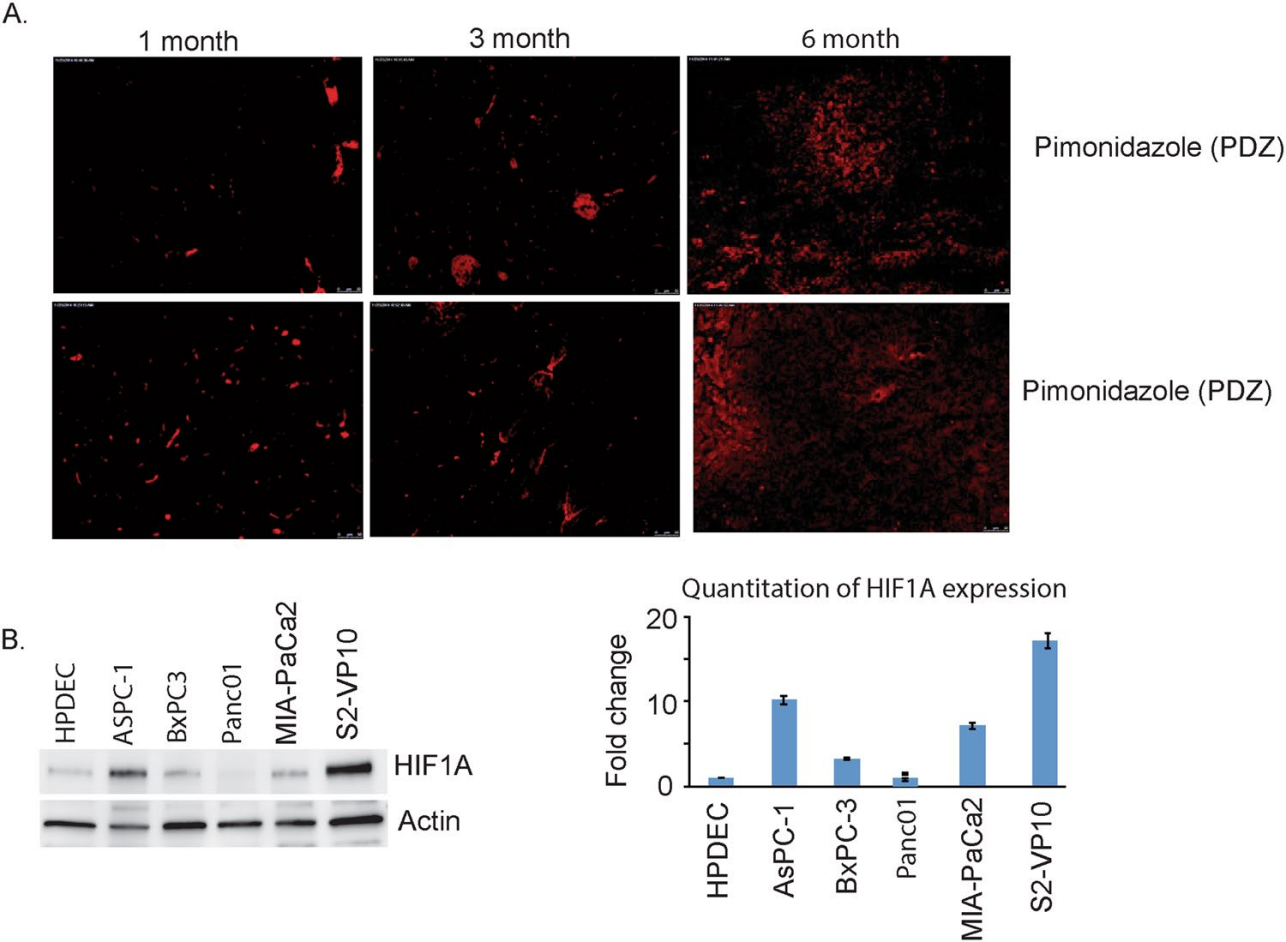
Pancreatic tumors are hypoxic. As the tumor in a KPC mouse progresses, there is increased hypoxic areas as seen by pimonidazole accumulation. Representative tumors from 6 KPC mice at different stages are shown (**A**). The cell lines show an increased expression of HIF1A protein compared to normal pancreatic ductal cells as seen in the western blot and quantitation (**B**).

The dense desmoplastic stroma in a pancreatic tumor compresses the blood vessels in the tumor resulting in a hypoxic tumor microenvironment. The Kras^G12D^-TP53^R172H^Pdx-Cre (KPC) mouse model of pancreatic cancer recapitulates the stages of pancreatic tumor progression as seen in patients^23^. 1 month tumor is typically representative of early tumor without manifestation of disease, 3 month represents a intermediate stage with extensive panIN lesions while 6 month or full tumor is an advanced tumor stage. Our results show that as the pancreatic tumors in KPC mice progressed, they showed increased areas of hypoxia within the tumor as visualized by accumulation of a hypoxia probe pimonidazole (PDZ)(Fig. 1A). Consistent with this, the expression of HIF-1α was found to be increased in the different pancreatic cancer cell lines at the protein level (Fig. 1B).

### Inhibition of hypoxia by Minnelide leads to accumulation of HIF1

Our previously published data indicate that Minnelide, a small molecule that has just completed a Phase 1 clinical trial and awaiting Phase 2, is able to decrease the stromal pressure and result in functional vasculature in a tumor^6^. Based on this observation, we hypothesized that Minnelide will have a profound effect on the hypoxic microenvironment of the tumor. To study this, we evaluated the hypoxia in Minnelide treated tumors using PDZ. Our results indicated that upon treatment of pancreatic tumors derived from patients (and xenografted in mice) or the genetically engineered KPC mice,Minnelide indeed decreased hypoxia in the tumor. Since pimonidazole (PDZ) accumulates in regions of low oxygen concentrations and the staining is not dependent of HIF1A protein levels, we next performed immunohistochemistry with anti-HIF1A on these tumors.

**Figure 2:**
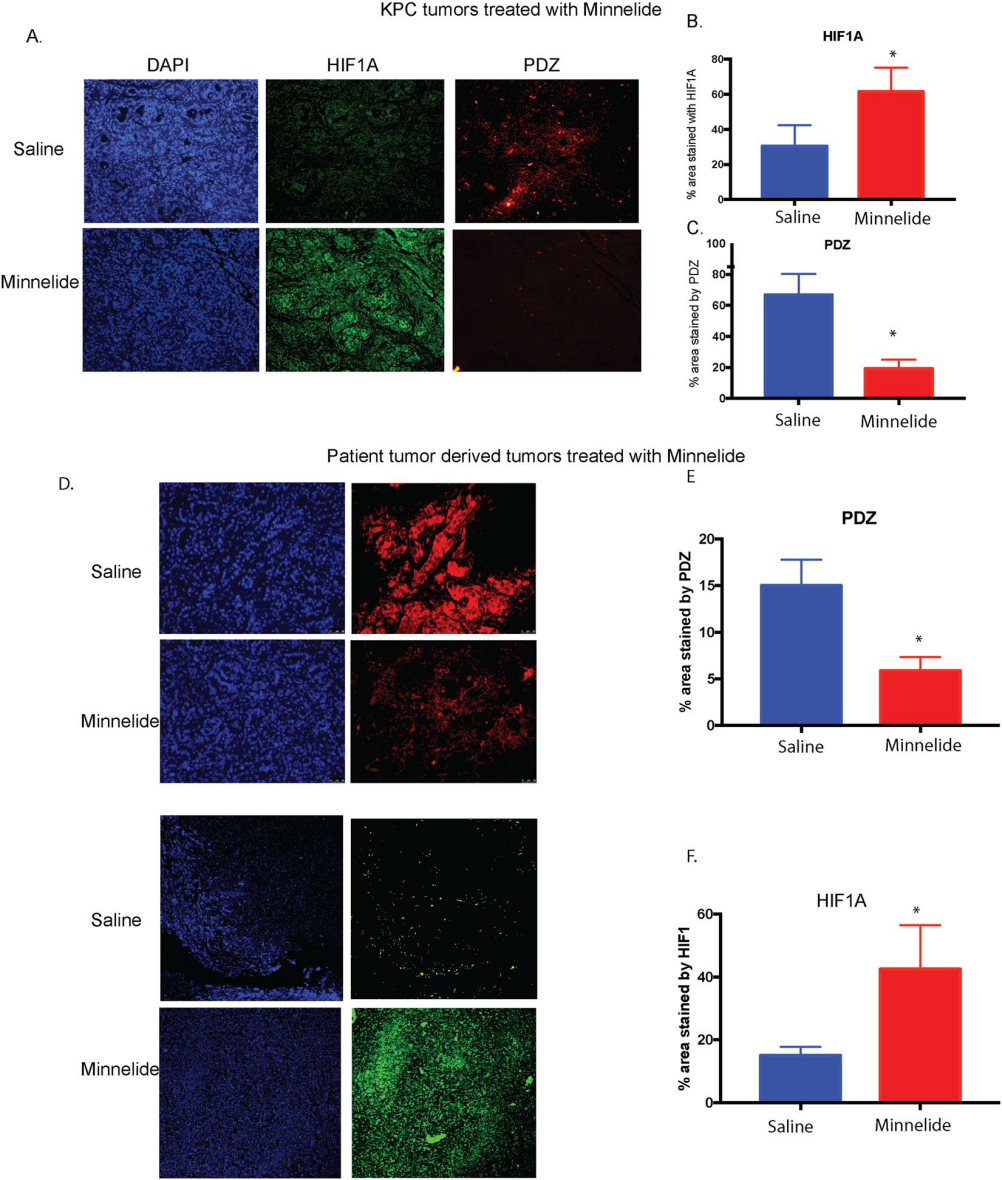
Treatment with triptolide pro-drug Minnelide increases accumulation of HIF1A protein in the tumors while decreasing hypoxia. KPC animals were treated with 0.42 mg/kg Minnelide and the tumors at the end of the treatment were stained for pimonidazole (PDZ) and HIF1A. representative images are shown (**A**), image from 5 animals is quantitated for HIF1A (**B**) along with PDZ (**C**). Similarly, patient tumor derived xenografts were treated with Minnelide 0.4 mg/kg for 20 days and the tumors were stained for PDZ and HIF1A. Representative tumor images are shown (**D**), image from 5 animals is quantitated for HIF1A (**B**) along with PDZ (**C**).

Interestingly, when stained with an anti-HIF1 antibody, our results showed that HIF-1α accumulated in the tumors (Fig. 2A–C). Similar accumulation of HIF-1α was also observed in patient tumor derived xenografts treated with Minnelide (Fig. 2D–F). This indicated that even though Minnelide was inhibiting the hypoxic state of the tumor, it was resulting in accumulation of HIF1A. Since hypoxia signaling is tightly regulated in a tumor cell at metabolic, post-translational and transcriptomic level, we next proceeded to evaluate each of this aspects following treatment with Minnelide and its active compounf triptolide.

### Accumulation of HIF-1α in pancreatic cancer upon inhibition of hypoxia by Minnelide is due to inhibition of metabolic pathways

**Figure 3:**
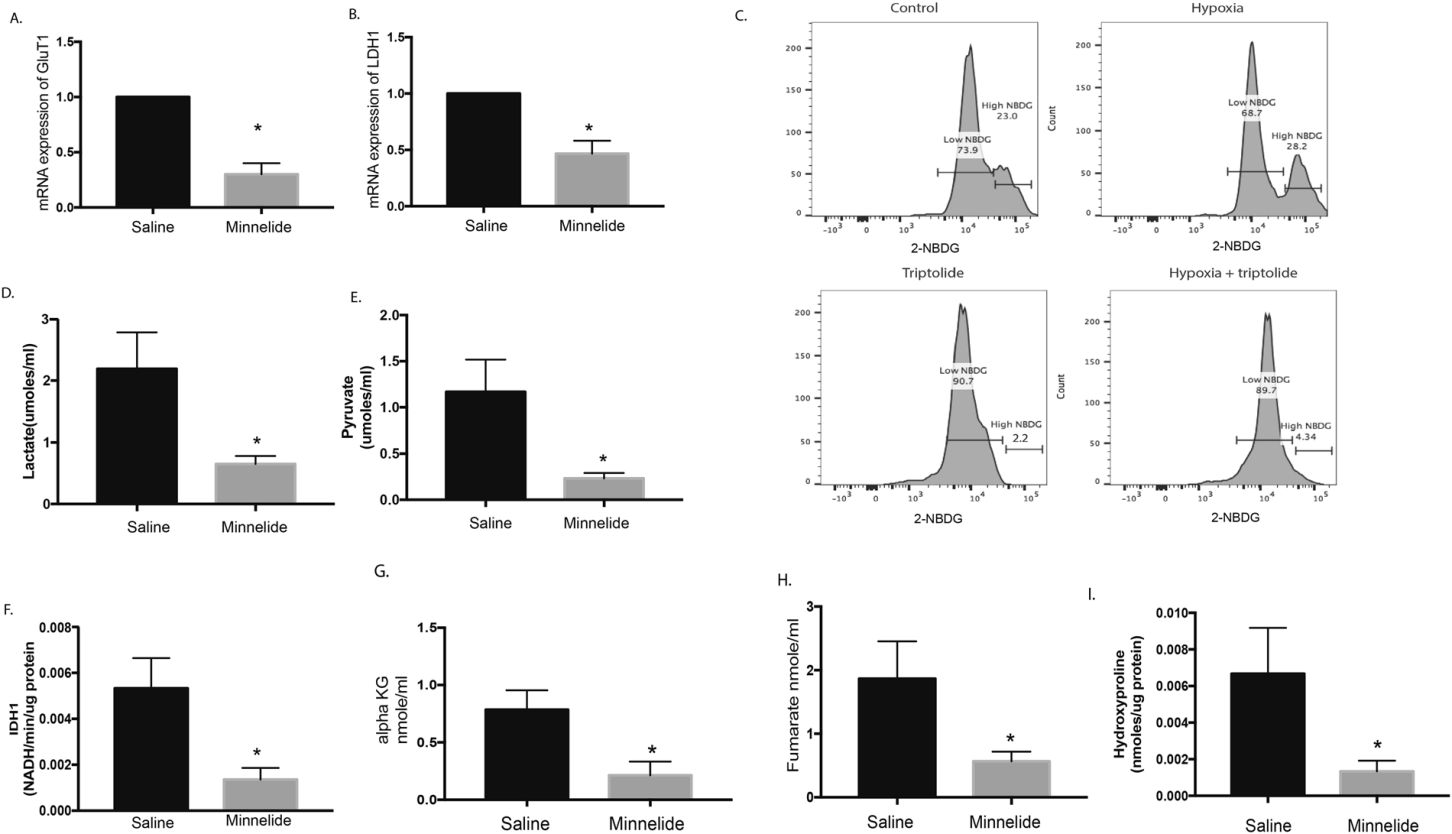
Downregulation of hypoxia by Minnelide affects metabolic pathways: Treatment with Minnelide (0.4 mg/kg) in a KPC animal decreased mRNA expression of GluT1 (**A**) and LDH1 (**B**). It also decreased the glucose uptake of the KPC001 cells *in vitro* following treatment with a low dose of 50 nM triptolide (**C**). In addition, the amount of lactate (**D**) and pyruvate (**E**) produced was also decreased. Treatment with Minnelide (0.4 mg/kg) in a KPC animals also decreased IDH1 activity (**F**) and alpha ketoglutarate formed (**G**). In addition, fumarate production was decreased following treatment (**H**). Minnelide also decreased the total hydroxyproline in the treated cells indicating a loss in PHD enzyme activity (**I**).

Hypoxia leads to increased glucose uptake by cells by upregulating the expression of glucose transporter gene GLUT1. In the absence of oxygen, the cancer cells preferentially undergo anaerobic glycolysis leading to production of lactate as a result of upregulated LDH1 activity. Both GLUT1 and LDH1 genes have hypoxia regulation element (HRE) sites in their promoter indicating that they are regulated by the oxygen status of the cell^24–26^. Treatment with Minnelide (*in vivo* on KPC tumors) decreased the mRNA expression of GLUT1 and LDH1 (Fig. 3A,B). Consistent with this, treatment of pancreatic cancer cells (MIA PaCa-2) with 50 nM triptolide (*in vitro* under hypoxia) showed less glucose uptake and less lactate produced (Fig. 3C,D). However, while it was expected that under decreased hypoxia, there would be increased pyruvate production, leading to a functional TCA cycle. However, we observed a distinct reduction in the pyruvate production (Fig. 3E).

Since pyruvate enters the TCA cycle in the presence of oxygen and is metabolized to produce ATP, we next estimated the activity of the TCA cycle enzyme IDH1 and the TCA cycle intermediates fumarate and alpha ketoglutarate. Treatment with Minnelide decreased activity of IDH1 along with a decrease in alpha ketoglutarate and fumarate (Fig. 3F,G,H).

It is well known that under normal oxygen levels, HIF1 is modified by the addition of a hydroxyproline by a group of enzymes: prolyl hydroxylases (PHD1-3). This enzyme in the presence of alpha keto-glutarate, from the TCA cycle, hydroxylates two proline residues in HIF-1α, which in turn facilitates its degradation. Under hypoxia, alpha ketoglutarate (αKG) production is reduced in the cells (owing to an anaerobic respiration), which contributes to accumulation of HIF-1α^27^. To confirm if a decrease in the alpha ketoglutarate led to decreased activity of PHDs, we next studied the effect of triptolide on total prolyl hydroxylation in pancreatic cancer cell lines. As hypothesized, treatment with triptolide resulted in a decreased total prolyl hydroxylation in pancreatic cancer cells (Fig. 3I). Since HIF-1α is one of the major proteins that is hydroxylated at the proline residue by the PHD, this indicated that triptolide also decreased the activity of this enzyme.

This evidence showed that the increase in the expression of HIF-1α in the tumor and in the cells in response to Minnelide treatment was due to the lack of co-factor αKG that was required for the activity of the enzymes PHD1-3. Thus, even though triptolide decreased hypoxia, it also affected the general metabolic pathways in the pancreatic cancer cells leading to decreased IDH1 activity, leading to a decreased synthesis of αKG, which in turn decreased PHD enzyme activity causing an accumulation of HIF-1α in the cells.

Since accumulation of HIF-1α can result from increased transcription of HIF-1α, we next evaluated the mRNA expression level of HIF-1α following triptolide treatment. Our studies further showed that treatment with triptolide decreased HIF-1α mRNA levels indicating that accumulation was not a result of increased transcription (Supplementary Figure 1).

HIF-1α can also accumulate if treatment with triptolide results in decreased proteosomal activity^28^. To study this, we assessed the activity of 20 S proteasome in the presence of triptolide. Our results showed that proteasomal activity following triptolide treatment was marginally decreased, indicating that this did not contribute significantly to stabilization of HIF-1α in the pancreatic cancer cells (Supplementary Figure 2).

### HIF1 transcriptional activity is inhibited by triptolide

**Figure 4:**
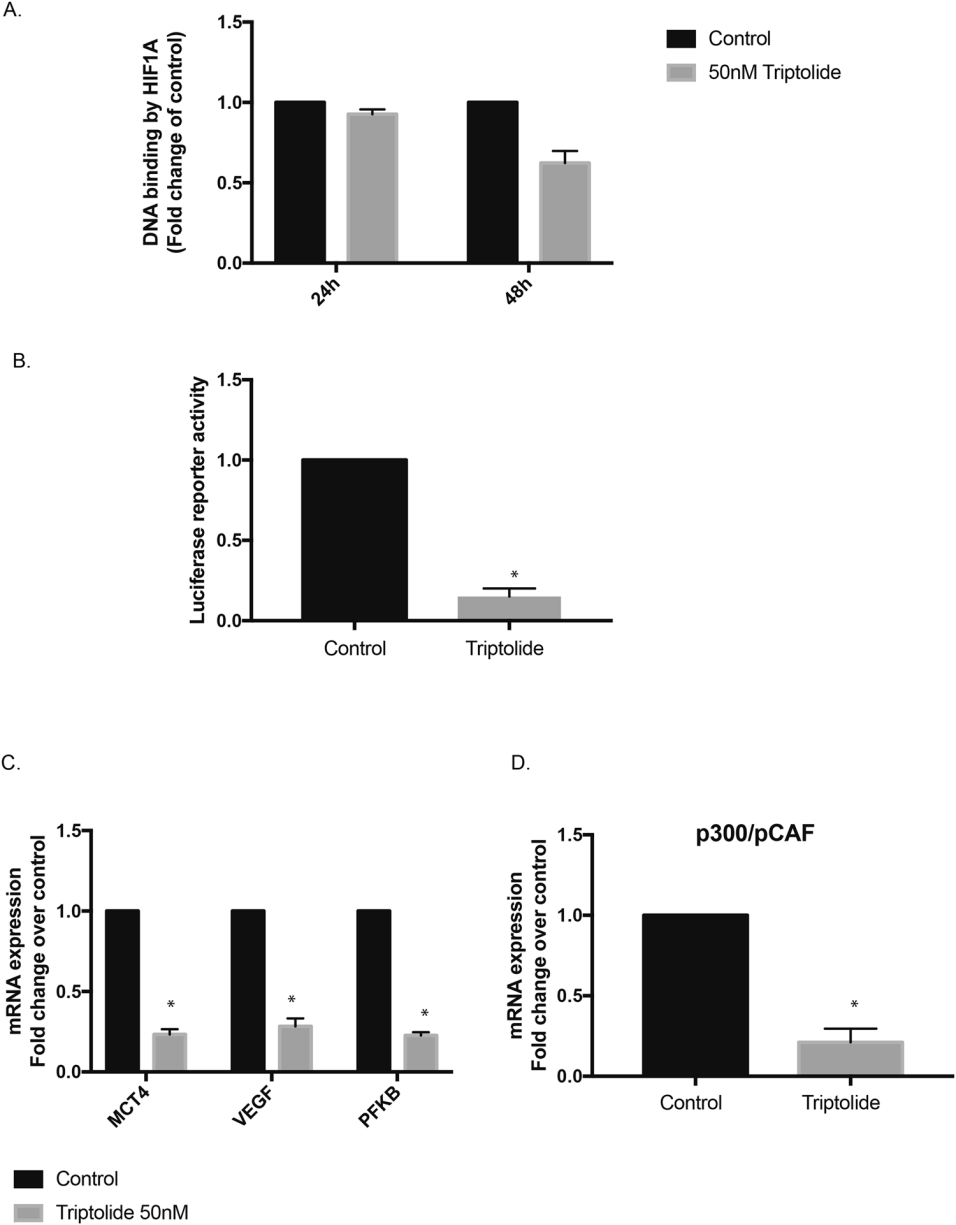
Triptolide decreases HIF1 transcriptional activity: Treatment with triptolide did not decrease the DNA binding activity of HIF1A in 24 or 48 h (**A**). However, treatment with triptolide did decrease the transcriptional activity in a luciferase reporter assay for HRE or hypoxia response element (**B**). Expression of VEGF, MCT4 and PFKB1 that are known downstream targets of HIF1A were decreased following treatment (**C**). Treatment with triptolide decreased p300 expression (**D**), indicating that triptolide was probably inhibiting the transcriptional complex formation in these cells.

To evaluate if the increased HIF-1α levels correlated with an increased HIF-1α transcriptional activity, we next performed a DNA binding assay. In this assay, the ability of HIF-1α to bind to the HRE was measured by an ELISA based assay. Our results showed that in the presence of triptolide (*in vitro*), there was no significant difference in the DNA binding in MIA PaCa-2 cells (Fig. 4A) or S2-VP10 cells (data not shown). To study if this correlated with the transcriptional activity of HIF-1α, we next performed a luciferase reporter assay. Our results showed that treatment of triptolide dramatically reduced the HIF-1α transcriptional activity as seen by decreased luminescence in the dual luciferase reporter assay (Fig. 4B).

To study if this further correlated with the expression of the downstream targets of HIF-1α, we next evaluated the expression of well-known HIF-1α targets (VEGF, PFKB1 and MCT4). Consistent with the decrease in the reporter activity, the downstream targets of HIF-1α were significantly decreased (Fig. 4C).

Effective transcriptional activity of HIF-1α is dependent on DNA binding and assembly of co-activators of transcription. Since we observed that there was no decrease in DNA binding and yet there was a decrease in transcriptional activity of HIF-1α, we next studied the expression of a co-activator of HIF-1α: p300. Our results showed that p300 expression was significantly downregulated by triptolide in pancreatic cancer cells (Fig. 4D).

### Inhibition of hypoxia leads to decreased oncogenic signaling

**Figure 5:**
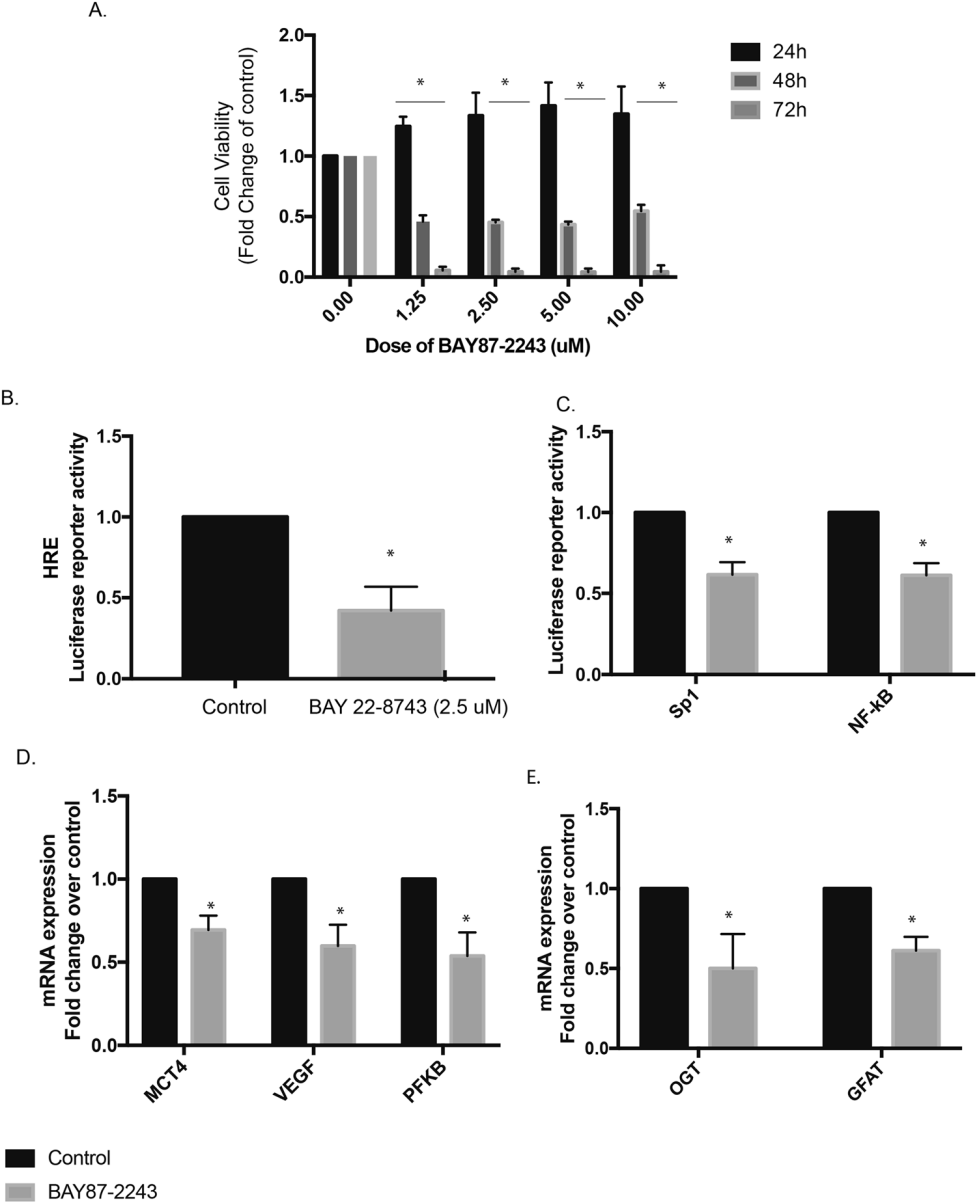
Inhibition of hypoxia leads to decreased oncogenic signaling. Treatment with BAY872243 decreased viability of pancreatic cancer cells (**A**) and HIF1A transcriptional activity by luciferase reporter assay (**B**). In addition, this also decreased the NF-kB and Sp1 transcriptional activity in these cells (**C**). Treatment with BAY 87-2243 also decreased the mRNA of VEGF, MCT4 and PFKB1, known targets of HIF1A (**D**). In addition, treatment with BAY87-2243 also decreased the expression of OGT and GFAT, the enzymes responsible for Sp1 and NF-kB transcriptional activity (**E**). All experiments were at least 3–5 times * indicates p-value < 0.05.

We and others have shown that Minnelide decreases oncogenic signaling by downregulating Sp1 and NF-kB in pancreatic tumors and causes regression^20^. To see if inhibition of hypoxia response led to decreased viability and oncogenic signaling, we inhibited hypoxia with chemical inhibitor BAY87-2243. Our results show that this compound decreased pancreatic cancer cell viability (Fig. 5A) and HIF-1α transcriptional activity (Fig. 5B), while having almost no effect on HIF-1α transcription levels (Supplementary Figure 3). Furthermore, a decrease in hypoxia also resulted in a decreased NF-kB and Sp1 activity (two transcription factors that are known to be affected by Minnelide) in these cells (Fig. 5C). In addition, treatment with this compound led to a decrease in expression of the downstream elements of HIF-1α (VEGF, MCT4, PFKB1) (Fig. 5D).

Since it is known that both Sp1 and NF-kB activity is dependent on the O-GlcNAc modification of the transcription factors, and this is dependent on the UDP-GlcNAc contect of the cells, we next studied the expression of OGT and GFAT expression in the presence of the hypoxia inhibitor. GFAT is the key enzyme that is involved in synthesis of UDP-GlcNAc which is used by OGT for modifying proteins. Our results showed that inhibition of hypoxia by BAY87-2243, resulted in a decrease in OGT and GFAT1 expression in pancreatic cancer cell lines (Fig. 5E) similar to that seen by Minnelide^20^.

### Inhibition of hypoxia by Minnelide leads to decreased stemness in pancreatic cancer cells

**Figure 6:**
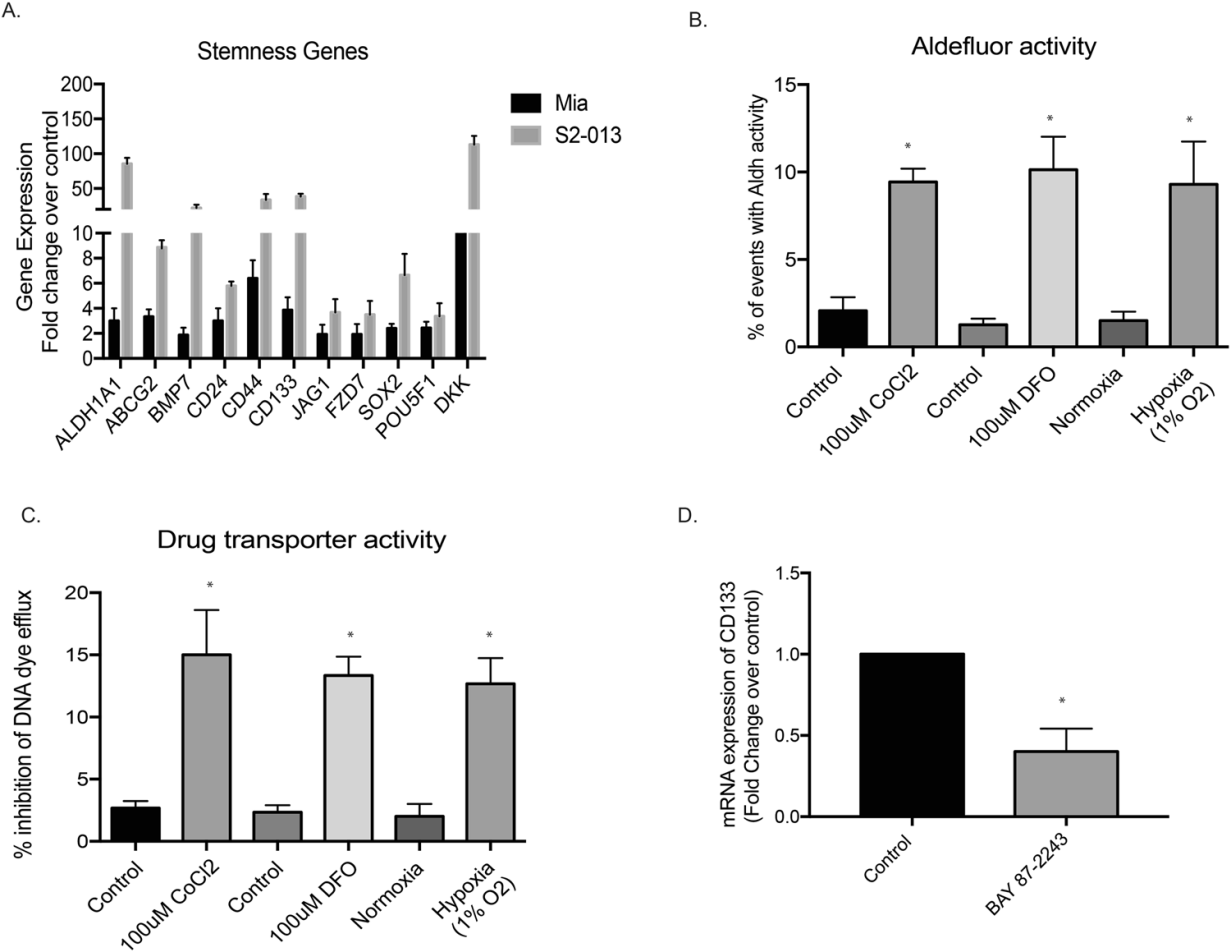
Hypoxia and stemness: Exposure of pancreatic cancer cells to hypoxia resulted in increased expression of stemness genes (**A**). In addition, Aldh activity, a known phenotypic marker of cancer stem cells, measured by Aldefluor was increased upon exposure to hypoxia or its chemical mimetics (**B**). In addition, upon activation of hypoxia mediated signaling, increased drug transporter activity was observed (**C**). Treatment with Minnelide decreased stemness in pancreatic cancer cells.

Previous results from our lab have shown that hypoxia leads to an enrichment of CD133^+^ cells (cancer initiating cell population). In the current study, our results showed that exposure to hypoxia by either growing cells in 1% O_2_ or by treating them with CoCl_2_ or DFO (a hypoxia mimetic), resulted in an increase in expression of stemness genes (Fig. 6A). Additionally, hypoxia also resulted in increased ALDH activity (Fig. 6B) and drug transporter activity (Fig. 6C) in these cells, indicating that hypoxia resulted in an increase in the stemness phenotype. To study if inhibition of hypoxia led to decreased stemness population, we next treated pancreatic cancer cells in hypoxia with BAY 87-2243. Our results showed that inhibition of hypoxia led to a decrease in the CD133+ cancer stem cell population.

## Discussion

The robust desmoplastic stroma of the pancreatic tumor in known to exert pressure on the tumor blood vessels resulting in their compression and creation of hypoxic niches. Other than correlating with increased resistance to therapy by preventing efficient delivery of drug to the tumor site, hypoxic niches often result in enrichment of cancer stem cells that contribute to poor prognosis of the tumor^17^. Previous studies from our group has shown that pancreatic tumor stroma and the cancer epithelial cells can be targeted by a water soluble prodrug of triptolide (a diterpene triepoxide), Minnelide^6^. Minnelide has recently completed a Phase I clinical trial and is currently awaiting a Phase II trial in pancreatic cancer patients^19^. Our studies have shown that Minnelide decreases the HA in the tumor stroma and prevents the crosslinking of collagen molecules, thereby relieving the stromal pressure on the collapsed tumor vasculature to open up blood vessels, rendering them functional blood vessels^6^. This indicated that treatment with Minnelide would result in decreased hypoxia in the pancreatic tumors and result in a downregulation of hypoxia mediated oncogenic signaling.

Results from our current study indicated that while treatment of Kras^G12D^-TP53^R172H^-PdxCre tumor bearing animals with 0.21 mg/kg Minnelide resulted in a decrease in hypoxia (as studied by pimonidazole, a hypoxia probe), it caused an accumulation of HIF-1α. HIF-1α is a tightly regulated protein. Under normal oxygen conditions, the cell undergoes glycolysis resulting in production of pyruvate, that enters the tricarboxylic acid cycle (TCA) before producing ATP as an energy currency via the electron transport chain. The intermediates of TCA cycle, alpha ketoglutarate (a product of the enzyme IDH1), acts a “oxygen sensor” and a cofactor for the enzymes prolyl hydroxylases (PHD). These enzymes hydroxylate the amino acid proline on HIF-1α, thereby marking them for degradation by a proteasome complex. Under hypoxia, majority of the glucose is utilized to produce lactate, which reduces the flux of the TCA cycle, leading to less production of alpha ketoglutarate^29^. This in turn, results in a decreased activity of PHD enzymes, leading to accumulation of HIF-1α. HIF-1α, then binds to the hypoxia responsive element (HRE) on the promoter of the responsive genes (like GLUT1, LDH1, VEGF) and upregulates their synthesis, which allows the cell to survive the hypoxic conditions^7–9^.

Our results showed that Minnelide on one hand decreased hypoxia, but caused an accumulation of HIF-1α. To determine why there was accumulation of HIF-1α in pancreatic cancer cells in spite of a significant decrease in hypoxia in the tumor, we next evaluated the transcription, translation and stability of HIF-1α in these cells. Our results showed that HIF-1α mRNA expression decreased after treatment with triptolide, while the protein levels increased (Supplementary Figure 1). This indicated that accumulation of HIF-1α was probably due to its decreased degradation. It is reported that triptolide may downregulate the 20 S proteasome activity in certain cancer cells. To study if accumulation of HIF-1α protein was because of the decreased proteasomal activity^30^, we studied the proteasome activity in pancreatic cancer cells following treatment with triptolide. Our results showed that proteasome activity was not significantly altered in the presence of triptolide (Supplementary Figure 2).

We next studied the physiological and bioenergetics aspects of inhibiting hypoxia in pancreatic cancer cells. We expected inhibition of hypoxia to lead to an increased TCA cycle activity (as this is fueled by oxygen). Interestingly, while treatment with Minnelide (*in vivo*) or triptolide (*in vitro*) decreased hypoxia (as studied by pimonidazole), and resulted in an expected decreased transcription of GLUT1 and LDH1, and a decrease in glucose uptake (Fig. 3), there was also a decrease in both lactate and pyruvate production under these conditions (Fig. 3). In addition, there was decreased accumulation of TCA cycle intermediates fumarate and α−ketoglutarate following treatment with Minnelide or its active drug triptolide. Since α ketoglurate is a co-factor that determines the activity of PHD enzymes in hydroxylating the proline residue in HIF-1α, decrease in α-ketoglutarate indicated a decreased activity by these enzymes (Fig. 3).

To study if triptolide affected the PHD enzyme expression along with its activity, we studied the expression level of PHD1-3. Our results showed that there was no significant decrease in the expression of the PHD enzymes following treatment with triptolide. This implied that the accumulation of HIF-1α was owing to a decreased hydroxylation of prolines by the PHD enzymes. Both binding of HIF-1α to the HRE element in the promoter of target genes as well as recruitment of co-activators regulate HIF-1α transcriptional activity^31, 32^. Our studies showed that triptolide did not alter the DNA binding of HIF-1α to the HRE regions, however luciferase reporter activity showed that triptolide decreased the transcriptional activity of HIF-1α leading to decreased synthesis of downstream targets (MCT4, LDH1). The HIF-1α subunit also contains two transactivation domains (TAD), which regulate HIF-1 target genes. CREB binding protein (CBP) and p300, two transcriptional co-activators of HIF-1, interact with the carboxy-terminal transactivation domain (C-TAD) of HIF-1α. Treatment with triptolide decreased the p300 mRNA, indicating that transcriptional activity of HIF-1α was decreased as triptolide decreased the mRNA of p300 (Fig. 4), thereby preventing the active HIF transcriptional complex to be formed^33^.

To study the effect of downregulated hypoxia mediated signaling, we next studied the effect of hypoxia inhibition on key pathways that are activated in pancreatic cancer. BAY87-2243 has been reported as an effective hypoxia inhibitor^34, 35^. Our studies showed that BAY87-2243 decreased viability of pancreatic cancer cell lines and also decreased the activity of Sp1 and NF-kB the two key oncogenic pathways (Fig. 5). In addition, inhibition of hypoxia by BAY-2243 also decreased the expression of OGT, a protein that glycosylates Sp1 and NF-kB to regulate their transcriptional activity.

Since hypoxia is known to enrich for cancer stem cells, we next studied the effect of inhibition of hypoxia on CD133^+^ cancer stem cell population, our results showed that downregulation of hypoxia and hypoxia mediated signaling decreased the CD133 expression level in pancreatic cancer cells.

## Conclusion

Hypoxia and hypoxia mediated signaling is among one of the most studied pathways in any cancer. Minnelide, a drug that has successfully completed Phase 1 trial and is awaiting Phase 2 trial has shown remarkable effect in downregulating a number of key pancreatic cancer pathways like Sp1 and NF-kB mediated signaling. In this study we show for the first time that all the downstream effects of Minnelide are being modulated by decreasing hypoxia and decreasing hypoxia mediated signaling. This is consistent with our previous finding that Minnelide depletes the stroma and renders blood vessels functional. This along with decreasing the hypoxia mediated oncogenic signaling has a profound effect on the viability of the tumor cells, including a decrease in stem cell population. This study is a step closer in understanding the mechanism of action of Minnelide that has shown a lot of promise in pre-clinical studies as well as in a Phase 1 trial against GI cancers.

## Electronic supplementary material

Supplementary Data
