# Supplementary material for: Inhibition of hypoxic response decreases stemness and reduces tumorigenic signaling due to impaired assembly of HIF1 transcription complex in pancreatic cancer
Source: Sci Rep. 2017 Aug 11;7:7872. doi: 10.1038/s41598-017-08447-3 (PMC5554238; doi:10.1038/s41598-017-08447-3)
Supplement: Supplementary file 1 — Supplementary Data [file 41598_2017_8447_MOESM1_ESM.pdf]

Inhibition of hypoxic response decreases stemness and reduces tumorigenic signaling due to impaired assembly of HIF1 transcription complex in pancreatic cancer

Olivia McGinn[1], Vineet K. Gupta[2], Patricia Dauer[1],Nivedita Arora[1], Nikita Sharma[2], Alice Nomura[1], Vikas Dudeja[2], Ashok Saluja[2] and Sulagna Banerjee*[2]

Supplementary Methods:

Proteasome Activity Assay

The 20S Proteasome Activity Assay Kit (Cayman Chemical) was used to determine proteasome activity *in vitro*. Briefly, cells were seeded in a 96-well plate at a density of 100,000 cells/well and grown overnight. Cells were lysed and the lysate was transferred to a black 96-well plate. A specific 20S substrate (SUC-LLVY-AMC) was added to the samples. When the substrate is cleaved by the 20S proteasome, a fluorescent product can be detected using an excitation wavelength of 360 nm and an emission wavelength of 480 nm.

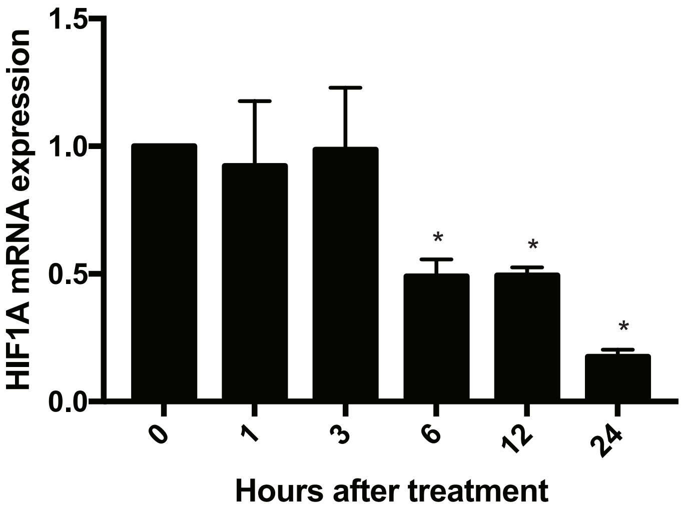

Supplementary Figure 1: HIF1A expression is decreased after treatment with 50nM triptolide for the indicated time.

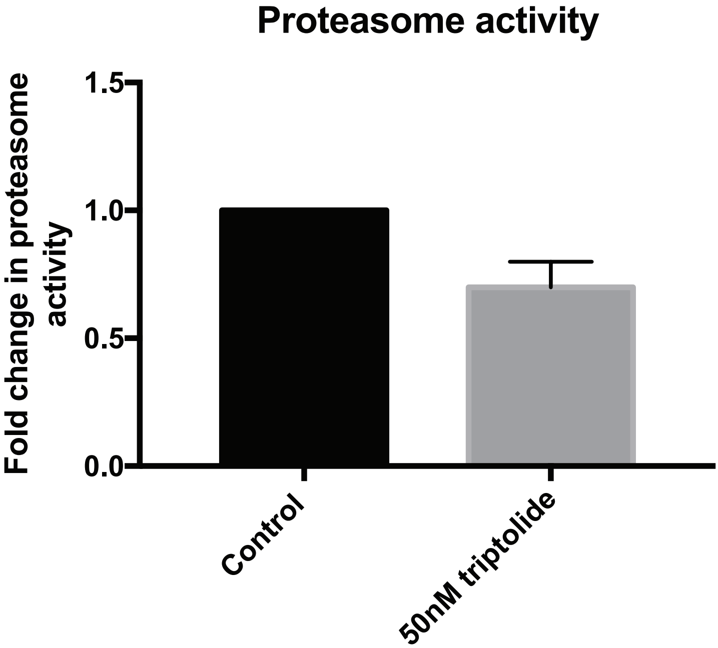

Supplementary Figure 2.

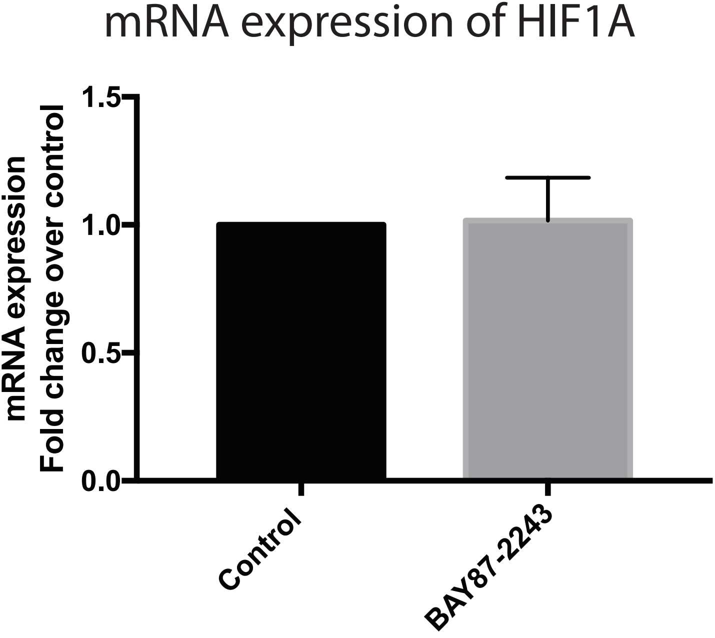

Supplementary Figure 3

Supplementary information
Full image of unedited gel showing expression of HIF1A in pancreatic cancer cell lines.

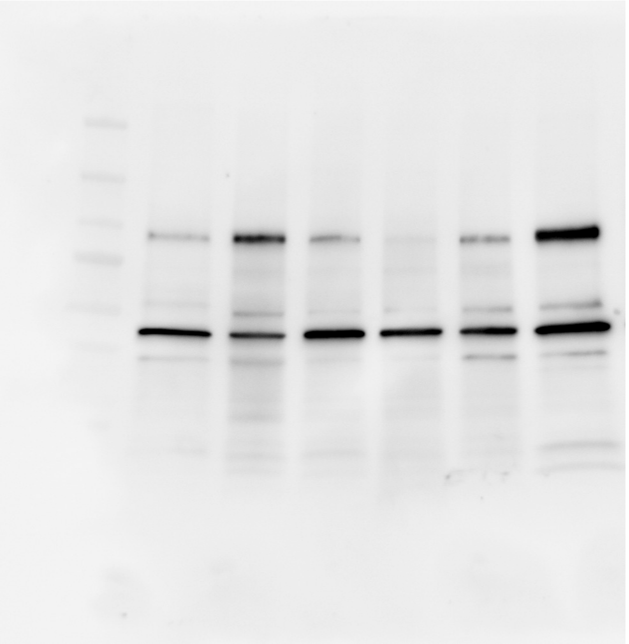

HIF1A (93KD)
Actin (42KD)
